# External Auditory Canal Eccrine Hidrocystoma and Equivocal Actinic Keratosis: A Rare Combination and Review of the Literature

**DOI:** 10.7759/cureus.105455

**Published:** 2026-03-18

**Authors:** Ved P Narang, Harshita Pai, Avneesh Kumar, Firas Al Hakim

**Affiliations:** 1 Otolaryngology - Head and Neck Surgery, St Helen's Hospital, Mersey and West Lancashire Teaching Hospitals NHS Trust, St Helen's, GBR; 2 Otolaryngology - Head and Neck Surgery, Whiston Hospital, Mersey and West Lancashire Teaching Hospitals NHS Trust, Whiston, GBR; 3 Otolaryngology, St Helen's Hospital, Mersey and West Lancashire Teaching Hospitals NHS Trust, St Helen's, GBR; 4 Otolaryngology, Whiston Hospital, Mersey and West Lancashire Teaching Hospitals NHS Trust, Whiston, GBR

**Keywords:** actinic keratosis, blue dome cyst, eccrine hidrocystoma, excision biopsy, premalignant lesion

## Abstract

Eccrine hidrocystoma is a rare benign cyst arising from the sweat glands, whereas actinic keratosis is a premalignant cutaneous lesion. Scheuermann’s kyphosis is a structural spinal disorder typically identified during adolescence. The coexistence of these conditions has not previously been reported. A patient in their early 70s presented with a blue, dome-shaped cyst located in the deeper portion of the external auditory canal. Surgical excision was performed, and histopathological examination revealed an eccrine hidrocystoma with equivocal features of actinic keratosis. The patient also had a known diagnosis of Scheuermann’s kyphosis. Although the hidrocystoma was completely excised, the premalignant potential of actinic keratosis necessitated close clinical follow-up. This rare case underscores the importance of comprehensive histopathological evaluation and vigilant monitoring of potential premalignant lesions. Awareness of such uncommon associations may aid clinicians in optimizing management strategies and ensuring appropriate long-term follow-up.

## Introduction

Eccrine and apocrine hidrocystomas are uncommon, benign, cystic lesions arising from the sweat glands and are most frequently encountered in the head and neck region [1]. Their occurrence within the external auditory canal is exceptionally rare, with fewer than 10 cases documented in the literature [2]. Clinically, these lesions may present as solitary (Smith type) or multiple (Robinson type) variants and have been reported with similar frequency in both sexes [3].

Eccrine hidrocystomas are typically small, measuring approximately 1-3 mm in diameter, and may gradually enlarge over time unless surgically excised or disrupted by trauma [3]. Multiple eccrine hidrocystomas are reported more frequently in women and tend to occur in areas prone to hyperhidrosis, particularly the periorbital and malar regions [3,4]. Associations have been described with Graves disease, Parkinson's disease, idiopathic craniofacial hyperhidrosis, and prolactinoma [4-6].

We report the case of a man in his early 70s with a solitary, well-circumscribed nodule measuring approximately 5-6 mm, located in the deeper portion of the external auditory canal. The lesion was detected incidentally during routine audiologic evaluation and was asymptomatic at presentation. This case highlights an unusual anatomic presentation of hidrocystoma. We aim to describe the clinical, radiologic, and histopathologic findings and to discuss management considerations. Meticulous microscopic examination is warranted on follow-ups, given the potential premalignant implications raised by the coexistence of equivocal actinic keratosis in this patient [7].

## Case presentation

A 72-year-old male patient was referred to the ENT clinic after the detection of a lump in the left external auditory canal during routine hearing screening. He was known to have long-standing Scheuermann’s kyphosis. The patient had trouble fitting a hearing aid on the affected side.

Examination under the microscope revealed a cystic lump measuring approximately 6 mm × 6 mm on the posterior canal wall. Initially, the patient declined examination under anaesthesia (EUA) but subsequently opted for excision after three months to exclude malignancy despite minimal symptoms apart from blockage due to wax accumulation. Audiometry demonstrated an age-related bilateral hearing loss consistent with presbycusis, and tympanometry showed normal middle ear compliance. 

**Figure 1:**
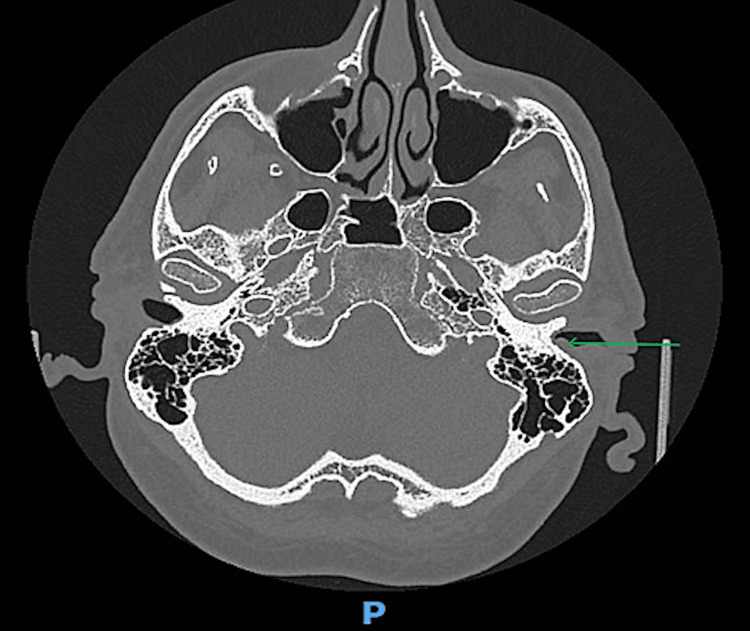
CT scan (axial view) of the temporal bone showing a 6 × 6 mm soft tissue lesion (green arrow) in the left external auditory canal without evidence of bony erosion

**Figure 2:**
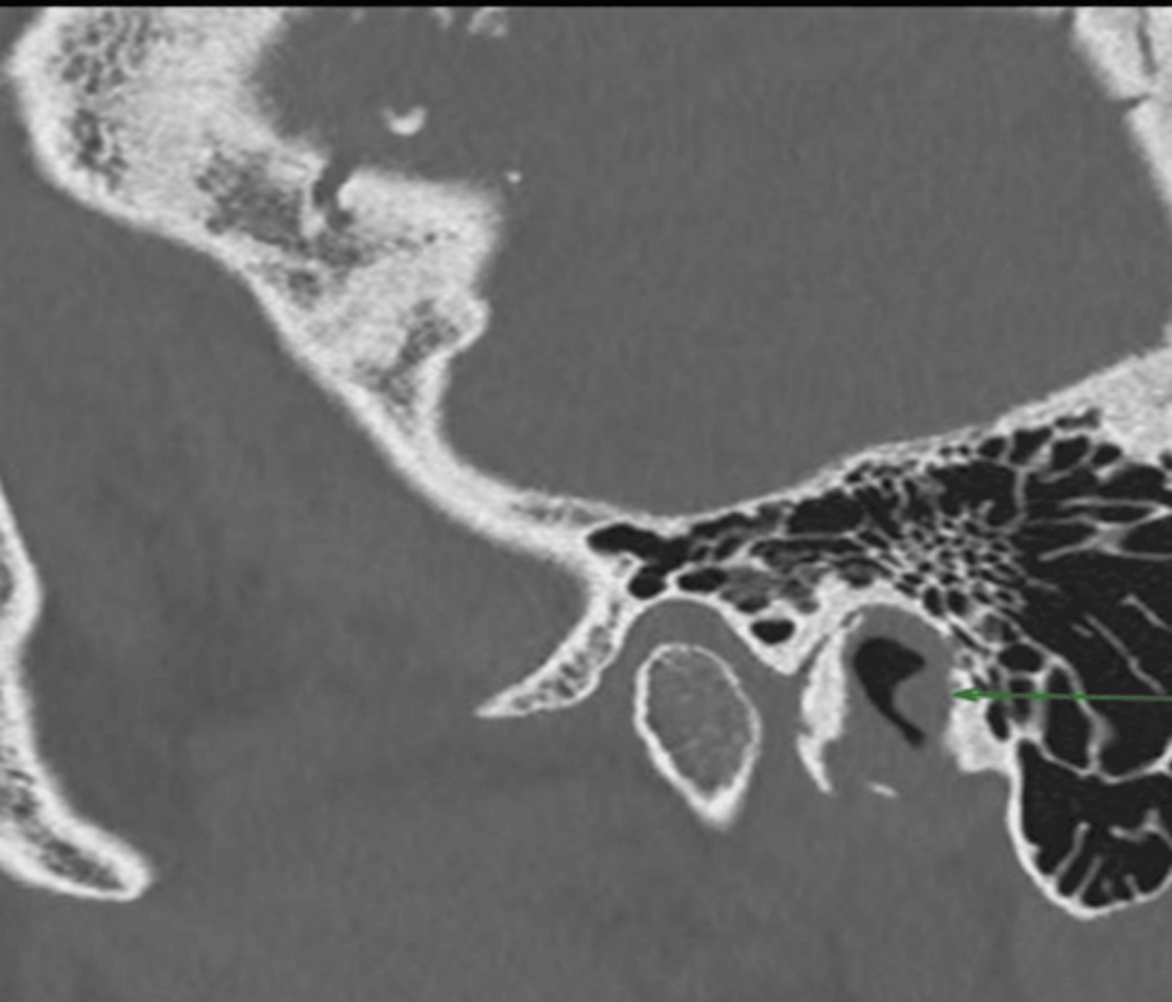
CT scan (sagittal view) of the left external auditory canal showing a 6 × 6 mm soft tissue lesion without bony erosion.

**Figure 3:**
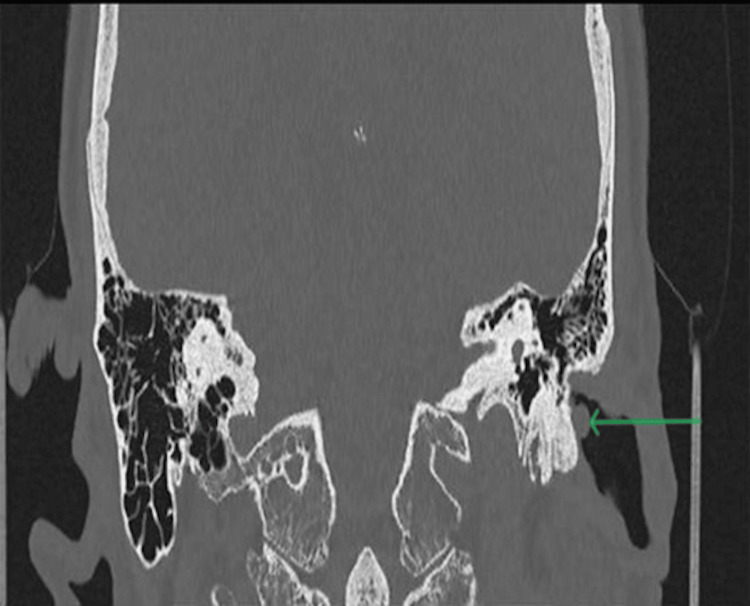
CT scan (coronal view) of the left external auditory canal showing a 6 × 6 mm soft tissue lesion without bony erosion.

CT scans revealed the presence of a soft tissue mass measuring 6 mm x 6 mm, over the posterior bony canal wall without any signs of bony erosions (Figures 1-3).

The lesion was excised with a 1-2 mm margin of clinically normal skin and deepened to the bone of the external auditory canal, including the periosteum. The excision specimen consisted of a bluish, dome-shaped cyst measuring 6 mm x 6 mm. Upon excision, the cyst contained mucinous secretion. Following the procedure, the canal was packed with a Bismuth Iodoform paraffin paste (BIPP) dressing, which was removed after three weeks, revealing a healthy canal wall

**Figure 4:**
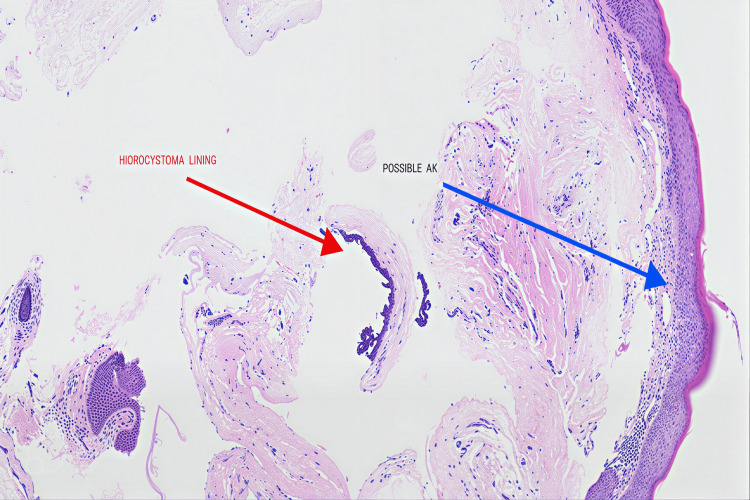
Histopathology (Hematoxylin and Eosin staining) of the cyst wall showing mostly denuded lining with focal areas of double-layered epithelium and papillary tufts, characteristic of hidrocystoma (red arrow). The overlying epidermis demonstrates mild parakeratosis with minimal atypia, suggestive but not definitive for actinic keratosis (blue arrow).

The histopathological examination was consistent with an eccrine hidrocystoma; however, the findings were not definitive for actinic keratosis. The histopathology team considered the features to be equivocal for actinic keratosis, a premalignant lesion (Figure 4).

## Discussion

Eccrine hidrocystomas typically present as slow-growing, bluish, dome-shaped cysts and are generally solitary outside periocular regions [1,2,8]. These can be multiple in ocular regions [4]. Differential diagnoses include ceruminous adenoma, epidermoid cyst, and other benign adnexal tumors of the canal [9].

Histopathological differentiation between eccrine and apocrine types depends on epithelial characteristics; eccrine variants exhibit a simple cuboidal epithelium with watery content, while apocrine lesions show decapitation secretion [3]. Our case uniquely revealed mucinous content on naked eye observation consistent with eccrine origin. Eccrine hidrocystomas are generally lined by a single layer of cuboidal epithelium and can be differentiated from apocrine hidrocystomas by the absence of a myoepithelial layer, papillary projections of the luminal secretory layer, and decapitation secretions [10].

The clinical similarity to malignant lesions makes surgical excision the preferred diagnostic and therapeutic intervention [4]. This case was managed with microscope-assisted excision, which provided effective visualization and removal of the lesion. Endoscopic excision methods have also been described as effective alternatives [4].

Though eccrine hidrocystomas are benign, the overlying equivocal actinic keratosis introduces premalignant considerations given its established potential for progression to squamous cell carcinoma [7]. Hence, regular follow-up is recommended for early identification of malignant transformation, although the ideal surveillance interval has yet to be established. The authors recommend a five-year surveillance period for lesions with histologically demonstrated premalignant potential, with follow-up every three months in the first year, every six months for the subsequent two years, and annually thereafter.

The patient had a long-standing history of Scheuermann’s kyphosis [11], which presented a challenge in achieving a comfortable and safe supine position on the operating table for both the patient and the surgical team. A review of the literature revealed no reported association between Scheuermann’s disease and eccrine hidrocystoma, along with equivocal actinic keratosis. The coexistence of these conditions in this patient, therefore, appears to be coincidental.

## Conclusions

This case highlights the importance of considering eccrine hidrocystoma in the differential diagnosis of external auditory canal masses, despite its rarity. Surgical excision remains the mainstay of treatment, with both microscopic and endoscopic approaches demonstrating favourable results.

The presence of associated equivocal actinic keratosis warrants careful surveillance, given the recognised premalignant potential of actinic keratosis. Accordingly, long-term follow-up is advisable to monitor for any evidence of progression or recurrence and to ensure optimal clinical outcomes. Further studies are needed to better define optimal surveillance strategies.
